# Evaluating the Diagnostic Potential of Myxovirus Resistance Protein 1 (MX1) and Myxovirus Resistance Protein 2 (MX2) As Biomarkers in Idiopathic Inflammatory Myopathies

**DOI:** 10.7759/cureus.102565

**Published:** 2026-01-29

**Authors:** Raghavee Neupane, Mustafa Haider, Perry Smith, Marc M Kesselman

**Affiliations:** 1 College of Medicine, Nova Southeastern University Dr. Kiran C. Patel College of Osteopathic Medicine, Fort Lauderdale, USA; 2 Rheumatology, Nova Southeastern University Dr. Kiran C. Patel College of Osteopathic Medicine, Fort Lauderdale, USA

**Keywords:** biomarkers, dermatomyositis, idiopathic inflammatory myopathies, interferon signature, mx1, mx2, myopathy

## Abstract

Idiopathic inflammatory myopathies (IIM) are a heterogeneous group of autoimmune muscle diseases characterized by proximal muscle weakness, systemic involvement, and high morbidity. Current diagnosis relies on clinical assessment, serology, and muscle biopsy, but challenges remain due to overlapping features and cases lacking clear biomarkers. Identifying molecular markers that reflect underlying disease pathways could improve diagnostic accuracy and patient stratification. This systematic review evaluates the diagnostic potential of myxovirus resistance protein 1 (MX1) and myxovirus resistance protein 2 (MX2), two interferon-inducible genes, as biomarkers in IIM and related conditions. A comprehensive literature search of PubMed, Embase, Web of Science, Ovid, and CINAHL (2015-2025) identified studies examining MX1 and/or MX2 in muscle disease. Eligible studies assessed diagnostic accuracy, sensitivity, specificity, or clinical relevance. Data extraction and risk of bias assessment followed the Preferred Reporting Items for Systematic Reviews and Meta-Analyses (PRISMA) and Joanna Briggs Institute guidelines. Evidence suggests that MX1 is consistently elevated in affected muscle tissue in IIM, particularly dermatomyositis, where it demonstrates high tissue specificity, though its expression is not exclusive to this subtype. Peripheral or blood-based MX1 measurements show promise but risk false positives and are influenced by disease activity, prior treatment, and timing of sampling. MX2 is consistently upregulated at the transcriptomic level, but protein-level data are limited, and its incremental diagnostic value over MX1 remains unclear. Sensitivity and specificity vary across studies, emphasizing the need for standardized protocols and further benchmarking against other interferon-inducible biomarkers or clinical tools. Overall, MX1 is a valuable tissue biomarker for interferon-mediated myopathies, with potential applications in diagnosis, disease activity assessment, and monitoring systemic progression. Future prospective, multicenter, and longitudinal studies are required to validate these markers, address current limitations, and determine their utility in routine clinical practice.

## Introduction and background

Idiopathic inflammatory myopathies (IIM) are a heterogeneous group of autoimmune disorders characterized by proximal muscle weakness and variable extramuscular involvement [1-3]. Up to 85% of patients with IIM have myositis-specific antibodies (MSAs), which strongly correlate with disease phenotypes and prognosis [4]. Major clinical subtypes include dermatomyositis (DM), polymyositis (PM), inclusion body myositis (IBM), amyopathic dermatomyositis (ADM), antisynthetase syndrome (ASyS), and immune-mediated necrotizing myopathy (IMNM) [1]. IMNM, often triggered by statins, is associated with anti-3-hydroxy-3-methylglutarylcoenzyme A reductase (anti-HMGCR) antibodies and persistent muscle weakness despite statin cessation [5]. IIM arises from genetic, medication-related, and environmental risk factors, involving both innate and adaptive immune responses [1,6,7]. Adult IIM subgroups, except ASyS and IBM, have a two to sevenfold increased risk of malignancies, particularly lung and breast, compared to the general population, with the risk highest one year before and after diagnosis [1].

First-line treatment includes high-dose glucocorticoids in combination with immunosuppressive drugs and immunoglobulin therapy, though standardized protocols for dosage and duration are lacking [1,4,8]. Ten-year survival rates range from 20% to 90%, with the main causes of death including malignancies, cardiovascular disease, and lung disease [1]. In North America, IIM affects an estimated 2.9 to 34 individuals per 100,000, with risk factors including viruses, smoking, and human leukocyte antigen (HLA) susceptibility [1].

Diagnosis remains challenging due to clinical heterogeneity and the absence of formal criteria. Clinicians rely on the European Alliance of Associations for Rheumatology/American College of Rheumatology (EULAR/ACR) classification criteria, combining clinical features, muscle biopsy, MRI patterns, and serology [1]. Standard laboratory markers, such as creatine kinase, aldolase, lactate dehydrogenase, and transaminases, are nonspecific, and approximately 20-30% of patients are seronegative [1,4,9]. While muscle biopsy is the gold standard for the diagnosis of IIM, MRI is increasingly used to guide sampling [4,10].

IIM subgroups differ in tissue involvement: IMNM and IBM primarily affect skeletal muscle, whereas DM and ASyS are multi-organ diseases [1]. Interstitial lung disease (ILD) occurs in up to 78% of IIM patients, especially in ASyS and DM. Anti-MDA5 antibodies are associated with an increased risk of severe respiratory complications [1]. Arthritis is common in ASyS, often mimicking rheumatoid arthritis, while dysphagia affects up to 60% of IBM patients [1]. MSAs are most reliably detected by immunoprecipitation, though it is a time consuming and not routine [1]. Indirect immunofluorescence has limited sensitivity due to weak staining of cytoplasmic antigens [1]. Conventional laboratory markers and MSAs have been previously studied. However, their sensitivity and specificity are limited, highlighting the need for novel interferon (IFN)-inducible biomarkers such as myxovirus resistance protein 1 (MX1) and myxovirus resistance protein 2 (MX2). Validated high-risk biomarkers, such as anti-MDA5 antibodies, can guide early aggressive therapy in patients at risk of ILD [6]. Early diagnosis and treatment guided by validated biomarkers are imperative to maintain improved health outcomes, as IIM is a progressive disease that causes irreversible tissue damage [1].

Type I IFN (IFN-I) pathway activation is a key molecular signature in DM, where it is most prominently expressed compared to other IIM subtypes. Early microarray studies in juvenile DM muscle showed upregulation of interferon-stimulated genes (ISGs) by over 100-fold compared to the controls [11], a finding later confirmed in adult DM, establishing the “interferon signature” as a distinguishing molecular feature [12]. This aligns with pathology, as perifascicular atrophy, the hallmark lesion of DM, is enriched with ISG expression, such as MX1, MX2, and ISG15 [13]. Innate immune activation, via toll-like receptors and plasmacytoid dendritic cells, drives IFN-I expression in muscle and skin [14,15]. IFN activity varies across IIM subsets, with DM largely driven by IFN-I, ASyS, and IBM by IFN-γ signatures, and IMNM showing little IFN activation [16-19]. Anti-MDA5-positive DM, prone to rapidly progressive ILD, exhibits the highest IFN-I scores [20,21]. These findings support IFN-I signaling as a key mechanism in DM and provide a rationale for IFN-inducible biomarkers.

MX1 (encoding MxA) and MX2 (encoding MxB) are IFN-inducible GTPases consistently upregulated in DM [22]. Integrated analyses show their expression alone distinguishes DM from controls with high accuracy (area under the curve (AUC) >0.90) [23]. MxA has the strongest protein level evidence as a diagnostic biomarker. Sarcoplasmic MxA staining demonstrates approximately 77% sensitivity and 100% specificity for DM, even without perifascicular atrophy [24,25]. Other IIMs, such as ASyS, IMNM, and IBM, rarely stain positive, highlighting their specificity [24]. By contrast, evidence for MX2 is primarily transcriptomic, co-upregulated with MX1 and may complement diagnostic panels [22,23,26]. Its nuclear localization has hindered assay development, but reproducible transcriptomic signals suggest potential utility once protein-level detection is standardized, e.g., via fluorescence in situ hybridization (FISH). Peripheral blood assays measuring MX1 are emerging, particularly in anti-MDA5 DM, where transcript levels correlate with disease activity, offering potential for non-invasive monitoring [21].

Despite the evidence, limitations exist. MxA sensitivity varies across studies with the biopsy timing, site, disease activity, and prior treatment [24,27]. MX2 lacks standardized protein-level assays, and its added diagnostic value over MX1 is unclear. MX1 is also elevated in other systemic autoimmune diseases, including lupus and systemic sclerosis, as well as during viral infections, potentially leading to false positives [28,29]. Comparisons of MX1 and MX2 with other IFN-inducible biomarkers, such as ISG15 or OAS family members, or clinical tools, such as autoantibody testing, MRI, or EMG, underscore current limitations in comparative and prospective data [30]. Therefore, prospective studies are needed to determine whether MX1 and MX2 can reduce diagnostic delays or misclassification.

This systematic review evaluates MX1 and MX2 as diagnostic biomarkers in IIM, focusing on their sensitivity, specificity, and clinical utility for early detection and disease differentiation.

## Review

Methods

A comprehensive literature search was conducted across Embase, Web of Science, Ovid (MEDLINE), PubMed, and CINAHL using keyword terms related to MX1 and MX2 (e.g., "MX1", "MX2", "myxovirus resistance protein 1", "myxovirus resistance protein 2"), inflammatory myopathies (e.g., "dermatomyositis", "polymyositis", "myositis", "muscle inflammation"), and diagnostic relevance (e.g., "biomarker", "diagnostic accuracy", "sensitivity", "specificity"). Boolean operators ("AND", "OR") were used to combine search terms appropriately. To ensure the recency of the articles, only English-language articles published between January 1, 2015, and July 1, 2025, were assessed. Titles and abstracts were screened using Rayyan (Rayyan Systems Inc., Cambridge, MA, USA), which supported duplicate removal and two-tier review (initial title/abstract screening followed by full-text evaluation) [31]. The search was limited to peer-reviewed, published studies. The Nova Southeastern University (NSU) library database was utilized to access databases and full-text articles.

Eligibility Criteria

The PICO framework guided our search strategy. The Population (P) included patients with confirmed or suspected muscle disease, including IIM. The Intervention/Index Test (I) was an assessment of MX1 or MX2 expression. Comparators (C), when available, included healthy controls or individuals with other neuromuscular or inflammatory conditions. The Outcomes (O) of interest were diagnostic performance metrics, including sensitivity, specificity, and overall diagnostic accuracy.

Studies were considered eligible if they investigated MX1 or MX2 in the context of muscle disease and assessed their diagnostic value, including sensitivity, specificity, diagnostic accuracy, or role in disease identification. Eligible study types included clinical trials, observational studies (cohort, case-control, or cross-sectional), case reports or case series, and relevant translational studies involving humans. Only studies involving patients with confirmed or suspected muscle disease were included.

Studies were excluded if they focused on MX1 or MX2 in contexts unrelated to muscle disease diagnosis, such as viral infections, oncology, or non-muscle autoimmune diseases. Articles were also excluded if they focused solely on treatment response, prognosis, or mechanistic pathways without assessing diagnostic performance. Reviews, editorials, commentaries, conference abstracts, animal-only studies, in vitro experiments, non-English publications, and articles without full-text access were also excluded.

Study Selection and Critical Appraisal of the Evidence

The initial search yielded 28 studies. After removing 10 duplicates, 18 unique articles remained. Of these, nine were excluded - five were conference abstracts and reviews, and four were unrelated to the topic. The remaining nine studies were screened through a blinded, two-tiered review process conducted by two independent reviewers, with a third reviewer resolving any discrepancies. Quality assessment was performed using the Joanna Briggs Institute Critical Appraisal Tools, which categorized risk of bias as low (>70%), moderate (50-70%), or high (<50%). Following this assessment, all nine articles that met the inclusion criteria were included in the final analysis.

**Figure 1:**
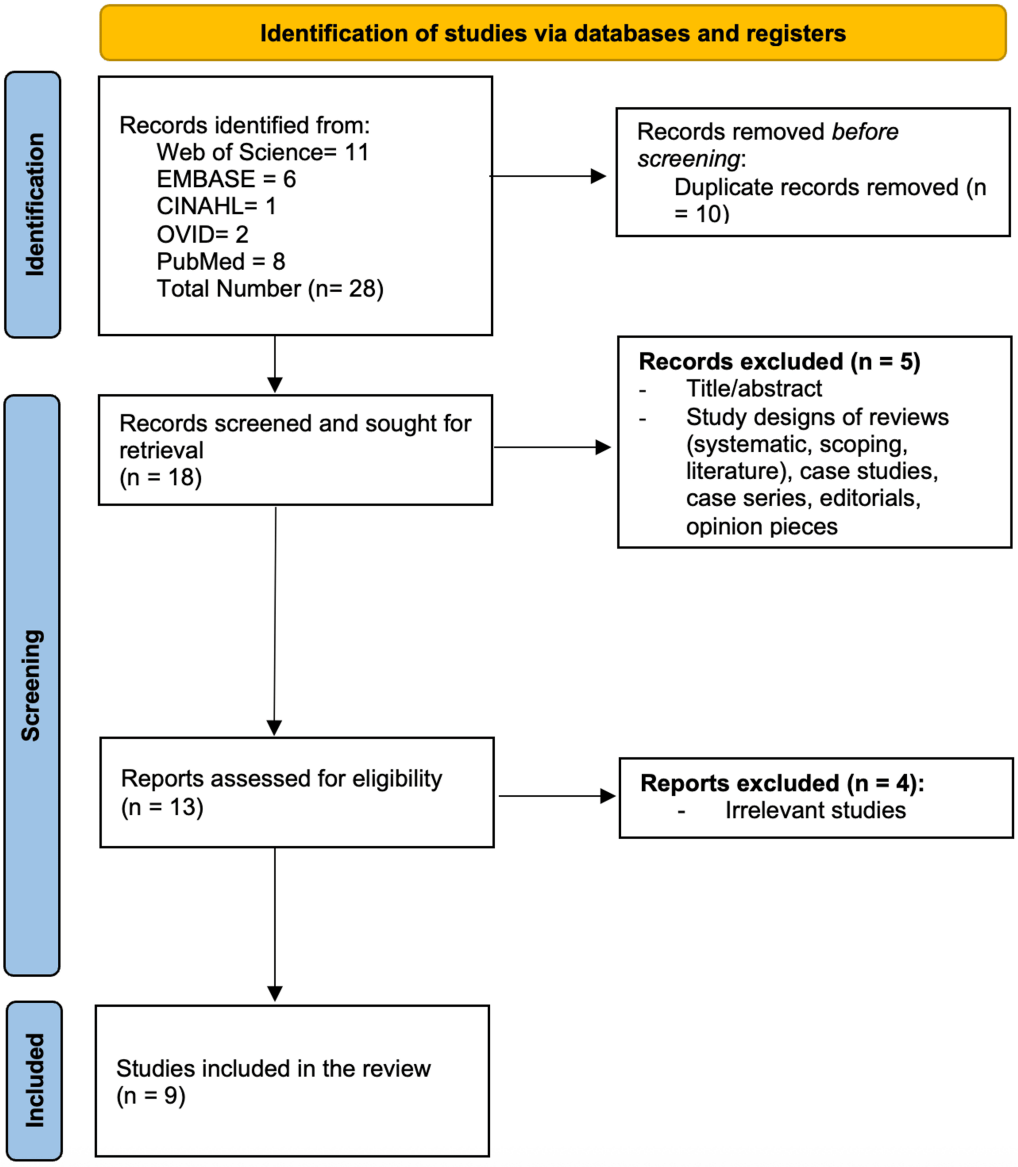
Preferred Reporting Items for Systematic Reviews and Meta-Analyses (PRISMA) flow diagram illustrating the study selection process

The review process adhered to Preferred Reporting Items for Systematic Reviews and Meta-Analyses (PRISMA) guidelines, and a PRISMA flow diagram was developed to illustrate the article selection process (Figure 1).

Results

Sample sizes ranged from individual patients to cohorts of up to 129 participants [32]. Most studies focused on patients with IIM, including DM, PM, and anti-synthetase syndrome. Four studies evaluated patients with fibrosing ILD, and one case report described MX1 expression in a patient with COVID-19-related myocarditis. Across cohorts, participants were predominantly female, representing as high as 75% of the study populations, and the mean age of participants generally ranged from the late 40s to early 60s.

MX1 was the most frequently investigated biomarker, examined in seven of the nine studies, either alone or in combination with MX2 or other IFN-stimulated genes [32-37]. Methods of assessment included quantitative reverse transcriptase PCR (qRT-PCR) of whole blood RNA (n=1) [33], immunohistochemistry (IHC) of muscle or myocardial biopsies (n=6) [32,34,36-38], protein microarrays (n=1) [35], and enzyme-linked immunosorbent assays (ELISA) for anti-MX1 autoantibodies (n=2) [35,38].

Across studies, MX1 expression was consistently upregulated in IIM compared with healthy and disease controls (n=9). DM cases demonstrated the strongest muscle staining, whereas non-IIM muscle tissues showed little to no expression. Elevated MX1 levels were correlated with markers of disease activity, including higher creatine kinase concentrations (n=1) and clinical muscle weakness (n=1). In the myocarditis case report, MX1 was markedly expressed in myocardial tissue, suggesting a broader role as a marker of IFN-mediated injury outside of skeletal muscle [36]. Anti-MX1 autoantibodies were identified in approximately 17% of patients with fibrosing ILD [35], particularly in those with non-specific interstitial pneumonia (NSIP). In these cohorts, the antibodies distinguished NSIP from idiopathic pulmonary fibrosis (IPF) with relatively high specificity but limited sensitivity.

MX2 was evaluated in only one of the included studies, all of which assessed its expression in the context of IFN-stimulated gene signatures [35]. Compared with MX1, MX2 expression was less consistently reported and generally appeared at lower levels across tissues. In DM cohorts, MX2 was modestly upregulated in both blood and muscle but did not show as strong an association with clinical disease activity as MX1. One study incorporating MX2 into a multi-gene panel found that it contributed to overall IFN-I scores but added limited discriminatory power when analyzed independently [34]. No studies examined anti-MX2 autoantibodies, and none specifically investigated their diagnostic or prognostic utility in IIM or related conditions.

Only three studies formally evaluated diagnostic or prognostic performance [35,37,39]. Among these, the presence of anti-MX1 antibodies showed potential in differentiating fibrosing ILD subtypes, and elevated MX1 expression was proposed as a biomarker of IFN-I pathway activation in IIM. Despite these findings, validation in larger, multi-center cohorts remains limited (Table 1).

**Table 1:** Characteristics and key findings of included studies on MX1/MX2 expression in disease ACR: American College of Rheumatology; Anti-NXP-2: Anti-nuclear matrix protein-2 antibody; Anti-SAE1: Anti-small ubiquitinlike modifier-1-activating enzyme antibody; Anti-TIF1γ: Anti-transcription intermediary factor 1 antibody; ARS: Aminoacyl-tRNA synthetase; aPAP: Autoimmune pulmonary alveolar proteinosis; AUC: Area under the curve; CD: Classification determinant; CD3/CD4/CD8/CD20/CD68/CD163: Cluster of Differentiation immune markers; CI: Confidence interval; CK: Creatine kinase; COVID: Coronavirus Disease; CT: Computed tomography; DLCO: Diffusing capacity of the lung for carbon monoxide; DM: Dermatomyositis; ELISA: Enzyme-linked immunosorbent assay; EULAR: European Alliance of Associations for Rheumatology; F: Female; Gal-9: Galectin-9 protein; GVHD: Graft-versus-host disease; HC: Healthy control; HLA: Human leukocyte antigen; HLA-DR: HLA class II DR isotype; IFN-I: Type I interferon; Ig: Immunoglobulin; IHC: Immunohistochemistry; IIM: Idiopathic inflammatory myopathy; IIP: Idiopathic interstitial pneumonia; ILD: Interstitial lung disease; IM: Inflammatory myopathy; IMNM: Immune-mediated necrotizing myopathy; INSIP: Idiopathic non-specific interstitial pneumonia; IPAF: Interstitial pneumonia with autoimmune features; IPF: Idiopathic pulmonary fibrosis; ISG: Interferon-stimulated gene; JDM: Juvenile dermatomyositis; M: Male; MAC: Membrane attack complex; MDA5: Melanoma differentiation-associated gene 5; MHC: Major histocompatibility complex; MRC-5: Medical Research Council cell strain 5; MSA: Myositis-specific autoantibody; MX1/MxA: Myxovirus resistance protein 1; MX2/MX3: Myxovirus resistance proteins 2 and 3; NSIP: Nonspecific interstitial pneumonia; OR: Odds ratio; RP-ILD: Rapidly progressive interstitial lung disease; RT-PCR: Reverse transcription polymerase chain reaction; Siglec-1: Sialic acid-binding Ig-like lectin 1

Author (Year)	Study Design	Size (M/F)	Mean Age (Years)	Medical History	Biomarker Measured (MX1/MX2)	Assay Method	Specimen Source	Expression Level Findings ↑/↓ in Disease vs. Control	Diagnostic Accuracy	Disease Activity/Severity	Conclusion
Tang et al., 2025 [33]	Cross-sectional observational single-center study	55 (75% F, 25% M) Subgroups: 25 MDA5(+) 19 ARS(+), 11 MSA(-)	46 ± 12	All had IIM. Subtypes: MDA5-positive dermatomyositis/clinically amyopathic DM, ARS-positive (anti-synthetase syndrome), or MSA-negative myositis. Many MDA5 positive has ILD	MX1	Quantitative RT-PCR for ISG gene expression in peripheral blood, Serum cytokines/chemokines were measured through multiplex assay (34-cytokine panel), Nailfold capillaroscopy	Whole blood for molecular analyses, and nailfold capillaries for assessment of microvascular changes.	Increased	Not mentioned	MX1 levels were higher in active than in clinically stable patients, suggesting upregulation is associated with greater disease activity.	MX1/IFN-I pathway activation correlates with microvascular damage in myositis, indicating Nailfold Videocapillaroscopy may serve as a noninvasive tool to monitor disease activity and interferon status.
Lee et al., 2022 [32]	Retrospective cross-sectional cohort study	129 (dermatomyositis n=66, polymyositis n=63). 66% F, 34% M; ILD + (38% of patients): 63% F ILD - (62% of patients): 68% F	Not mentioned	All patients had IIM (dermatomyositis or polymyositis). 38% had ILD confirmed by chest CT	MX1, other markers assessed included T-cell (CD3, CD4, CD8), B-cell (CD20), macrophage (CD68, CD163) markers, and MHC class I and II	IHC in formalin-fixed muscle biopsy sections	Muscle biopsy tissue (quads and delts)	Increased	Not mentioned	In univariate analysis, MX1-positive muscle cells were associated with ILD (OR ≈1.89, 95% CI 1.16–3.16, p=0.012). However, in multivariate logistic regression (adjusting for CD4 and MHC/HLA expression), MX1 did not remain an independent predictor of ILD (adjusted OR ≈1.5, p=0.15). Instead, HLA-DR expression on muscle fibers was the strongest independent predictor of ILD. No analyses correlate MX1 with muscle weakness or CK levels reported.	ILD in myositis is linked to stronger muscle immune activation, shown by more MX1 in inflammatory cells and HLA-DR on myofibers, highlighting a connection between muscle inflammation and lung involvement.
Ngo et al., 2024 [34]	Retrospective case series	56 (F:M ~ 3:1)	49.7 ± 16.1	All patients were diagnosed with IIM. The cohort included immune-mediated necrotizing myopathy (IMNM, 58.9%), dermatomyositis (23.2%), overlap myositis (8.9%), anti-synthetase syndrome (5.4%), inclusion body myositis (1.8%), and polymyositis (1.8%).	MX1/2/3	IHC on frozen muscle biopsy tissue (quads and delts)	Frozen muscle biopsy tissue (quads and delts)	Increase in MX1/2 expression was confined to dermatomyositis	Not mentioned	Nearly all patients had either upregulated MHC-I or membrane attack complex deposition; adding Mx did not significantly improve sensitivity due to its low frequency. No sensitivity/specificity was given specifically for Mx1/2/3	Combining multiple immunomarkers increases diagnostic yield (e.g., MHC-I + MAC detected 96% of IIMs), and IFN-induced Mx protein is a useful indicator of dermatomyositis muscle pathology.
Hamano et al., 2017 [35]	Nested case-control study	Cohort 1 (discovery, n=48): IPF (9/1), Idiopathic nonspecific interstitial pneumonia (INSIP) (4/4), autoimmune pulmonary alveolar proteinosis (aPAP) (4/6), Sarcoidosis (1/9), HC (8/2) Total = 54.17% M, 45.83% F Cohort 2 (validation, n=114): Anti-MX1+ (9/11), Anti-MX1- (70/24) Total = 69.29% M, 30.71% F	Cohort 1 (n=48): IPF: 62.9 years, INSIP: 58.5 years, aPAP: 49.3 years, Sarcoidosis: 58.9 years, HC: 49.9 years; Cohort 2 (n=114): MX1+ 75 years, MX1- mean: 73 years	Chronic fibrosing interstitial pneumonias: IPF, INSIP, aPAP, and sarcoidosis, were included to identify disease-specific autoantibodies across the ILD spectrum. Many patients met the criteria for interstitial pneumonia with autoimmune features	Anti-MX1 autoantibody	Protein microarray (antigen array). ELISA was used in a larger cohort to quantitatively measure anti-MX1 (of IgG, IgA, and IgM isotypes) and anti-ARS autoantibodies	Serum	Increase in anti-MX1 in ~17% of fibrosing IIPs - restricted to INSIP/autoimmune ILD, 0% in IPF	Anti-MX1 is found in a minority of NSIP cases (~17% of fibrosing ILD) and never in IPF, making it very good at ruling out IPF but only catching some NSIP cases.	Anti-MX1 marks a specific NSIP/autoimmune-type ILD group and helps distinguish it from IPF. Anti-MX1 doesn't predict severity as measured by DLCO, and is a unique antibody not seen with other myositis antibodies.	Anti-MX1 may serve as a diagnostic marker for fibrosing ILD, highly specific for non-IPF, present in ~17% of cases, and could help subclassify lIPs.
Kinoshita et al., 2023 [36]	Case report	1 M	49	Diabetes, Graves' disease, dyslipidemia, COVID-19, Hypertension, Complete atrioventricular block, and Cardiogenic shock	MX1	IHC testing	Myocardial biopsy	Increase	Not mentioned	Testing for dermatomyositis autoantibodies, anti-SAE1, anti-MDA5, anti-TIF gamma, and anti-NXP-2, predicts COVID severity.	Myocardial biopsy shows a perifascicular staining pattern of MxA, which suggests a strong pathological association with dermatomyositis
Liang et al., 2021 [38]	Cross-sectional and longitudinal studies	103 F/51 M/30 controls	49.0/47.0 (only median provided)	Dermatomyositis, Immune-mediated necrotizing myopathy	Gal-9	ELISA, IHC staining	Subject blood serum, MRC-5 fibroblasts from American Type Culture Collection, lung biopsy	Increase in Gal-9	Not mentioned	Gal-9 levels tend to increase with disease activity in anti-MDA5 DM and RP-ILD.	However, individual Gal-9 values cannot precisely quantify disease severity.
Ghang et al., 2024 [37]	Case control	129 IIM/73 controls	Not mentioned	Idiopathic inflammatory myopathies	CD3, CD4, CD8, CD20, CD68, CD163, MX1, MHC class I, MHC class II, HLA-DR	IHC staining	Muscle biopsy	Increase in CD163 and MHC Class I	AUC = 0.953, 0.961 (first is algorithms w/o IIM-histopathological features, second is with)	Both algorithms were able to identify 94.1% of patients who were diagnosed with IIM but did not meet the 2017 EULAR/ACR criteria for IIMs	Compared to histopathologic examination alone, the developed algorithms using CD163 and MHC Class I muscular expression had improved diagnostic accuracy
Lerkvaleekul et al., 2022 [39]	Prospective multicentre study	21 JDM/15 Healthy controls/7 Duchenne Muscular Dystrophy (controls for type I ISG expression and Siglec-1 expression on monocytes only)	8.1/11.4	Juvenile dermatomyositis (JDM)	Siglec-1, type 1 IFN	Multiplex immunoassay	Blood	Increase in Siglec-1	AUC = 0.87	Increased Siglec-1 expression "correlates with clinical disease activity", but without a quantitative relationship	Siglec-1 is a highly sensitive biomarker for risk of requiring treatment intensification
Shiota et al., 2022 [40]	Case report	1 F	46	Myelodysplastic syndrome, Acute GVHD with skin involvement	C5b9, MxA, MHC-II	IHC staining	Muscle biopsy	Increase in MxA and MHC-II	Not mentioned	Biopsy showed necrotic and regenerative fibers, interstitial fibrosis, and lymphocytic infiltrate, predominately CD8+ T cells. Expression of C5b9, MHC-II, and MxA has potential for diagnostic criteria of cGVHD-related IM.	This is the first reported case of cGVHD-related IM, showing increased MxA and MHC-I| on biopsy, consistent with the known immune pattern of cGVHD.

The methodological quality and risk-of-bias assessment of the included studies, conducted using the Joanna Briggs Institute Critical Appraisal Tools, is summarized in Table 2.

**Table 2:** Risk-of-bias assessment of included studies using the Joanna Briggs Institute tools

Author (Year)	JBI Tool Used	Risk of Bias
Tang et al., 2025 [33]	Checklist for Analytical Cross Sectional Studies	Low
Lee et al., 2022 [32]	Checklist for Cohort Studies	Low
Ngo et al., 2024 [34]	Checklist for Case Series	Low
Hamano et al., 2017 [35]	Checklist for Case Control Studies	Low
Kinoshita et al., 2023 [36]	Checklist for Case Reports	Low
Liang et al., 2021 [38]	Checklist for Analytical Cross Sectional Studies	Low
Ghang et al., 2024 [37]	Checklist for Case Control Studies	Moderate
Lerkvaleekul et al., 2022 [39]	Checklist for Cohort Studies	Low
Shiota et al., 2022 [40]	Checklist for Case Reports	Low

Discussion

In this systematic review, we synthesized evidence on the role of MX1 and MX2 in IIM. The findings reinforce the central role of the IFN-I pathway in IIM and highlight MX1 as a reproducible and biologically grounded biomarker, particularly in DM [31-33,36]. Across heterogeneous study designs and assay platforms, MX1 was consistently upregulated in affected muscle and blood, correlating with markers of disease activity, including creatine kinase, muscle weakness, and inflammation [32]. These findings highlight MX1 as a clinically relevant biomarker for assessing disease activity and guiding treatment, particularly in IIM cases lacking serologic markers.

Sensitivity of MX1 as a biomarker may be influenced by disease phase and timing of tissue sampling. Activation of the IFN pathway is the most pronounced early in DM, prior to the development of extensive fibrosis or fatty replacement. As a result, MX1 expression may be most informative during early or preclinical diagnosis, when conventional histopathologic findings are subtle or absent. If confirmed in prospective studies, MX1 could enable early diagnosis and support the timely initiation of immunomodulatory therapy, thereby reducing the risk of irreversible muscle damage.

MX1 may also help bridge between tissue-based and blood-based diagnostics. Although muscle biopsy remains the diagnostic gold standard, it is invasive and may be nondiagnostic depending on sampling site and disease stage. Similarly, serologic testing for MSAs is clinically useful but remains negative in up to 30% of patients. In this context, MxA IHC provides a direct tissue-based measure of IFN activation and demonstrates high specificity, even in cases lacking classic histologic features. This suggests that MX1 could serve as a complementary diagnostic marker, particularly in seronegative or diagnostically ambiguous cases.

Beyond skeletal muscle, MX1 expression has been implicated in systemic manifestations. Anti-MX1 autoantibodies have been detected in patients with fibrosing ILD, and MX1 expression has been reported in COVID-19-associated myocarditis, suggesting broader relevance as a marker of IFN-driven injury [34,35]. By contrast, MX2 has been less well studied. Evidence suggests modest upregulation in DM, with weaker associations with disease activity than MX1 and limited independent diagnostic or prognostic value [33]. No studies evaluated anti-MX2 antibodies, leaving their clinical significance uncertain.

Limitations of the current literature include small, single-center studies and heterogeneity in assay methods, ranging from quantitative reverse transcription polymerase chain reaction (qRT-PCR) to IHC and serologic testing [31-36]. Few studies systematically evaluated diagnostic accuracy (e.g., sensitivity, specificity, or area under the curve (AUC)) or prognostic value [34,36,38], but none examined the cost-effectiveness or feasibility of integrating MX1 or MX2 testing into clinical practice. Importantly, longitudinal studies are lacking, limiting conclusions about whether MX1 can track disease progression or predict therapeutic response over time. Standardization remains a major barrier to the widespread adoption of MX1 testing. Variability in immunohistochemical protocols, antibody clones, scoring thresholds, and biopsy site selection likely contributes to inter-study heterogeneity. Establishing consensus guidelines for MX1 assessment, similar to those developed for myositis-specific autoantibodies, will be critical before MX1 can be incorporated into routine diagnostic workflows. Multi-center validation using harmonized protocols should therefore be a priority for future research [24,25,27].

Beyond diagnosis, MX1 may have important implications for precision medicine in inflammatory myopathies. As targeted therapies directed against IFN signaling pathways continue to emerge, reliable biomarkers of pathway activation are needed to guide patient selection and therapeutic response. MX1 expression could potentially identify patients most likely to benefit from IFN-targeted or Janus kinase-inhibitor therapies, thereby avoiding unnecessary exposure to broad immunosuppression in patients without active IFN signaling. This positions MX1 not only as a diagnostic biomarker, but also as a candidate predictive biomarker for treatment stratification [41].

Taken together, MX1 emerges as a promising biomarker of IFN pathway activation in IIM, with potential applications in diagnosis, disease monitoring, and stratification of systemic involvement such as ILD [31-34]. However, its clinical utility remains preliminary, and MX2 is even less characterized.

## Conclusions

This review highlights MX1 as a consistent marker of IFN-I pathway activation in IIM and related conditions. Across multiple studies, MX1 expression was elevated in muscle and blood, correlated with disease activity, and showed potential for identifying systemic involvement, such as ILD. The presence of anti-MX1 autoantibodies further suggests a role in stratifying fibrosing lung disorders, although sensitivity remains limited. In contrast, evidence for MX2 is sparse, with only modest upregulation reported and little independent diagnostic or prognostic value. Overall, current findings support MX1 as a promising biomarker for diagnosis, disease monitoring, and potential therapeutic stratification in IFN-mediated diseases. However, the evidence remains preliminary, and widespread clinical adoption will require validation in larger, multicenter cohorts with standardized methodologies. Future work should also clarify the clinical significance of MX2 and assess whether combined IFN signatures can provide added value beyond MX1 alone.

## References

[1] Lundberg IE, Fujimoto M, Vencovsky J (2021). Idiopathic inflammatory myopathies. Nat Rev Dis Primers.

[2] Zhao Q, Hu Q, Meng S (2023). Metabolic profiling of patients with different idiopathic inflammatory myopathy subtypes reveals potential biomarkers in plasma. Clin Exp Med.

[3] Zubair AS, Salam S, Dimachkie MM, Machado PM, Roy B (2023). Imaging biomarkers in the idiopathic inflammatory myopathies. Front Neurol.

[4] Ashton C, Paramalingam S, Stevenson B, Brusch A, Needham M (2021). Idiopathic inflammatory myopathies: a review. Intern Med J.

[5] Tiniakou E (2020). Statin-associated autoimmune myopathy: current perspectives. Ther Clin Risk Manag.

[6] Yoshifuji H (2015). Biomarkers and autoantibodies of interstitial lung disease with idiopathic inflammatory myopathies. Clin Med Insights Circ Respir Pulm Med.

[7] Khoo T, Lilleker JB, Thong BY, Leclair V, Lamb JA, Chinoy H (2023). Epidemiology of the idiopathic inflammatory myopathies. Nat Rev Rheumatol.

[8] Aggarwal R, Charles-Schoeman C, Schessl J (2022). Trial of intravenous immune globulin in dermatomyositis. N Engl J Med.

[9] Lu X, Peng Q, Wang G (2015). Discovery of new biomarkers of idiopathic inflammatory myopathy. Clin Chim Acta.

[10] Benveniste O, Goebel HH, Stenzel W (2019). Biomarkers in inflammatory myopathies-an expanded definition. Front Neurol.

[11] Tezak Z, Hoffman EP, Lutz JL (2002). Gene expression profiling in DQA1*0501+ children with untreated dermatomyositis: a novel model of pathogenesis. J Immunol.

[12] Greenberg SA, Pinkus JL, Pinkus GS (2005). Interferon-alpha/beta-mediated innate immune mechanisms in dermatomyositis. Ann Neurol.

[13] Salajegheh M, Kong SW, Pinkus JL (2010). Interferon-stimulated gene 15 (ISG15) conjugates proteins in dermatomyositis muscle with perifascicular atrophy. Ann Neurol.

[14] Cappelletti C, Baggi F, Zolezzi F (2011). Type I interferon and Toll-like receptor expression characterizes inflammatory myopathies. Neurology.

[15] Wong D, Kea B, Pesich R (2012). Interferon and biologic signatures in dermatomyositis skin: specificity and heterogeneity across diseases. PLoS One.

[16] Bolko L, Jiang W, Tawara N, Landon-Cardinal O, Anquetil C, Benveniste O, Allenbach Y (2021). The role of interferons type I, II and III in myositis: a review. Brain Pathol.

[17] Gasparotto M, Franco C, Zanatta E (2023). The interferon in idiopathic inflammatory myopathies: different signatures and new therapeutic perspectives. A literature review. Autoimmun Rev.

[18] Pinal-Fernandez I, Casal-Dominguez M, Derfoul A (2019). Identification of distinctive interferon gene signatures in different types of myositis. Neurology.

[19] Greenberg SA (2010). Type 1 interferons and myositis. Arthritis Res Ther.

[20] Li M, Zhang Y, Zhang W (2023). Type 1 interferon signature in peripheral blood mononuclear cells and monocytes of idiopathic inflammatory myopathy patients with different myositis-specific autoantibodies. Front Immunol.

[21] Qian J, Li R, Chen Z, Cao Z, Lu L, Fu Q (2023). Type I interferon score is associated with the severity and poor prognosis in anti-MDA5 antibody-positive dermatomyositis patients. Front Immunol.

[22] Jeong HN, Lee TG, Park HJ, Yang Y, Oh SH, Kang SW, Choi YC (2023). Transcriptome analysis of skeletal muscle in dermatomyositis, polymyositis, and dysferlinopathy, using a bioinformatics approach. Front Neurol.

[23] Xiao L, Xiao W, Lin S (2021). Ten genes are considered as potential biomarkers for the diagnosis of dermatomyositis. PLoS One.

[24] Uruha A, Allenbach Y, Charuel JL (2019). Diagnostic potential of sarcoplasmic myxovirus resistance protein A expression in subsets of dermatomyositis. Neuropathol Appl Neurobiol.

[25] Uruha A, Nishikawa A, Tsuburaya RS (2017). Sarcoplasmic MxA expression: a valuable marker of dermatomyositis. Neurology.

[26] Wang X, Hu H, Yan G, Zheng B, Luo J, Fan J (2024). Identification and validation of interferon-stimulated gene 15 as a biomarker for dermatomyositis by integrated bioinformatics analysis and machine learning. Front Immunol.

[27] Waisayarat J, Wongsuwan P, Tuntiseranee K, Waisayarat P, Dejthevaporn C, Khongkhatithum C, Soponkanaporn S (2023). Sarcoplasmic myxovirus resistance protein A: a study of expression in idiopathic inflammatory myopathy. J Inflamm Res.

[28] Huijser E, van Helden-Meeuwsen CG, Groot N (2019). MxA is a clinically applicable biomarker for type I interferon activation in systemic lupus erythematosus and systemic sclerosis. Rheumatology (Oxford).

[29] Assassi S, Mayes MD, Arnett FC (2010). Systemic sclerosis and lupus: points in an interferon-mediated continuum. Arthritis Rheum.

[30] Tanboon J, Uruha A, Stenzel W, Nishino I (2020). Where are we moving in the classification of idiopathic inflammatory myopathies?. Curr Opin Neurol.

[31] Ouzzani M, Hammady H, Fedorowicz Z, Elmagarmid A (2016). Rayyan-a web and mobile app for systematic reviews. Syst Rev.

[32] Lee JS, Ghang B, Choi W (2022). Expression of inflammatory markers in the muscles of patients with idiopathic inflammatory myopathy according to the presence of interstitial lung disease. J Clin Med.

[33] Tang M, Shi J, Pang Y (2025). Nailfold videocapillaroscopy findings are associated with IIM subtypes and IFN activation. Arthritis Res Ther.

[34] Ngo DQ, Le ST, Phan KH (2024). Immunohistochemical expression in idiopathic inflammatory myopathies at a single center in Vietnam. J Pathol Transl Med.

[35] Hamano Y, Kida H, Ihara S (2017). Classification of idiopathic interstitial pneumonias using anti-myxovirus resistance-protein 1 autoantibody. Sci Rep.

[36] Kinoshita H, Kurashige T, Fukuda T (2023). The impact that myocarditis for post-acute COVID-19 syndrome may be dermatomyositis-like myocarditis: a case report. Heliyon.

[37] Ghang B, Nam SH, Choi W (2024). Expression of CD163 and major histocompatibility complex class I as diagnostic markers for idiopathic inflammatory myopathies. Arthritis Res Ther.

[38] Liang L, Zhang YM, Shen YW (2021). Aberrantly expressed galectin-9 is involved in the immunopathogenesis of anti-MDA5-positive dermatomyositis-associated interstitial lung disease. Front Cell Dev Biol.

[39] Lerkvaleekul B, Veldkamp SR, van der Wal MM (2022). Siglec-1 expression on monocytes is associated with the interferon signature in juvenile dermatomyositis and can predict treatment response. Rheumatology (Oxford).

[40] Shiota T, Eura N, Hasegawa A, Kiriyama T, Sugie K (2022). Pathological features of inflammatory myopathy as a manifestation of chronic graft-versus-host disease after allogeneic bone marrow transplantation. Neuropathology.

[41] Ladislau L, Suárez-Calvet X, Toquet S (2018). JAK inhibitor improves type I interferon induced damage: proof of concept in dermatomyositis. Brain.

